# Controls on fluvial sediment evacuation following an earthquake-triggered landslide: Observations from LiDAR time series

**DOI:** 10.1126/sciadv.adi5560

**Published:** 2024-09-04

**Authors:** Jon Tunnicliffe, Jamie Howarth, Chris Massey

**Affiliations:** ^1^ School of Environment, University of Auckland, Auckland, New Zealand.; ^2^ School of Geography, Environment and Earth Sciences, Victoria University of Wellington, Wellington, New Zealand.; ^3^ GNS Science, Te Pū Ao Avalon, Lower Hutt, New Zealand.

## Abstract

Catastrophic sediment overloading of mountain streams in response to coseismic landsliding causes river systems to fundamentally reorganize their morphology and sediment transporting characteristics, influencing sediment yields, bedrock incision, and the coupling between erosion and tectonics. A sequence of 13 airborne LiDAR surveys of an alpine tributary of the Hāpuku River, New Zealand, reveals patterns of sediment mass balance change over 5 years following delivery of 6.6 million cubic meters of landslide debris during the 2016 magnitude 7.8 Kaikōura earthquake. The surveys reveal how mountain river systems modulate catastrophic sediment deliveries to their lower reaches through sediment storage, evolution of channel morphology, and armoring of the bed. Variations in valley width contribute to the delay and diffusion of the seismically induced disturbance “wave” as it moves across river process domains. The landslide sediment train remnants may persist for longer than the return time of their triggering mechanism, leading to a long-lived hiatus in bedrock incision in this tectonically active mountain catchment.

## Teaser

Thirteen LiDAR surveys over 5 years show a time lapse of river buildup and evacuation in the wake of the 2016 Kaikōura earthquake.

## INTRODUCTION

Bedrock incision by mountain rivers is a fundamental control on landscape evolution in active mountain belts because it counters tectonic uplift, forms valleys by steepening hillslopes, and sets the long-term bedrock landsliding rate where hillslopes are at threshold angles (*1*–*5*). Erosion of rivers into bedrock is mediated by the rate of energy expended by flow on the riverbed (stream power) and the episodic supply of sediment from hillslopes (*6*, *7*). Sediment provides tools for incision when the transport capacity of the river exceeds sediment supply, but, when sediment supply exceeds the transport capacity, sediment covers the bed, greatly reducing erosion into bedrock (*8*, *9*). Large earthquakes may trigger tens of thousands of landslides on hillslopes that instantaneously deliver millions of cubic meters of sediment to mountain rivers, substantially altering their sediment transport regime and morphodynamics (*10*–*12*). The overloading of rivers with coseismic landslide-derived sediment causes rivers to shift from a supply-limited, potentially incisional mode, to one dominated by aggradation that can exceed tens of meters (*13*). The residence time of this sediment, relative to the return time of earthquake-induced landsliding (*14*, *15*), is a rate-limiting factor on bedrock incision. Therefore, characterizing the processes that drive the evacuation of sediment and the time frame over which it occurs is important for understanding landscape evolution in active mountain belts (*16*–*18*). These processes also moderate flood conveyance and the severity of flooding, which, in turn, affects post-earthquake resilience and recovery (*19*).

Observations of rivers responding to coseismic landslide-derived sediment show that the initial aggraded deposit can be long-lived, persisting for decades to centuries (*20*), although direct and quantitative observations are exceedingly rare. Analysis of satellite imagery combined with limited field surveys showed that rivers in the epicentral area of the 2008 moment magnitude (*M*_w_) 7.9 Wenchuan earthquake, China, were still aggrading a decade after the earthquake (*13*). Retrospective analysis using historical air photos and field surveys of rivers from the epicentral area of the 1929 *M*_w_ 7.9 Murchison earthquake, New Zealand, was used to infer that they were still responding to landslide-derived sediment 50 years after the earthquake (*20*). Reconstructions using the sediment record preserved in alluvial deposits demonstrated that rivers around Pokhara, Nepal, may still be responding to massive aggradation triggered during several strong medieval earthquakes (*21*, *22*). While these studies demonstrate that the response of mountain rivers to landslide sediment overloading may be long lived, they do not constrain sediment evacuation time frames or the processes controlling them.

Modeling has been used to estimate sediment evacuation times of coseismic landslide sediment and to explore the governing processes (*11*, *13*). Modeling of sediment evacuation times after the 1999 *M*_w_ 7.3 Chi-Chi earthquake in Taiwan was based on the volume of landslide sediment supplied to rivers and river transport capacity and predicted century scale evacuation times that may be longer than the recurrence interval of landslide-triggering earthquakes (*13*). However, more detailed numerical simulations that capture dynamic changes to channel hydraulic geometry show that the progressive evolution of the river into a narrower, incised channel may promote more effective evacuation of coseismic landslide sediment within years to decades, allowing mountain streams to return rapidly to supply limited conditions (*11*). However, these models ignore a broad spectrum of interacting factors that could control the response of mountain streams to earthquake-induced landsliding (*19*).

The evacuation time of major landslide debris accumulations in mountain valleys may be governed by factors such as the grain size composition of the landslide material (*23*, *24*), the intensity and sequencing of mobilizing flood flows, and local valley gradient and width. The caliber and sorting of the landslide-derived material may favor the development of a coarse-grained armor, through selective accumulation of boulder-sized material, for instance (*25*–*27*). It has been shown that the presence of boulders can strongly influence the evolution of montane river systems, potentially forming knickpoints and locally increasing slope. The intermittency of flooding may condition the structuring of the bed over time (*28*–*31*), potentially modulating the threshold stress for particle entrainment. The availability of accommodation space for a transient surplus of sediment is likely to moderate the passage of material through a montane drainage network as well (*32*–*34*). Thus, determining evacuation times of coseismic landslide sediment requires improved understanding of the spectrum of factors that control the response of mountain streams to earthquake-induced landsliding (*19*). Ideally, this is achieved using real-world observations that resolve the coevolution of sediment supply conditions, channel morphology, bed texture, and the mass balance associated with the transient storage and transfer of materials in mountain valley streams responding to sediment overloading (*35*).

**Fig. 1.**
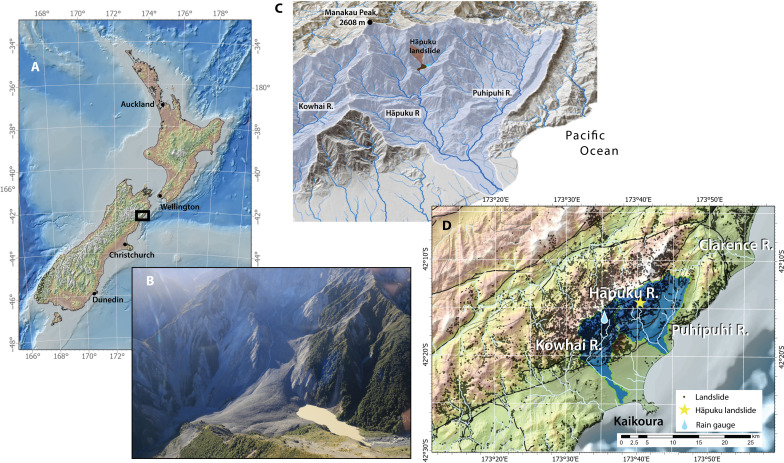
Location of the Hāpuku River and landslide, in the Seaward Kaikōura Ranges, Canterbury Region, New Zealand. The study area extends from the Seaward Kaikōura Range to the coast, near the town of Kaikōura (**A**). A lake was impounded upstream of the landslide deposit (**B**). The landslide is roughly 13 km from the river ocean outlet (**C**). Dots on the map in (**D**) show the locations of roughly 6000 seismically activated landslides in the immediate vicinity.

Here, we examine the linkages between sediment supply conditions, channel morphology, grain size, and valley width in a steep (>0.1 m m^−1^) alpine valley immediately after the *M*_w_ 7.8 14 November 2016 Kaikōura earthquake and over a period of 5 years. This study period covers an episode of extreme sediment overloading and subsequent evacuation. We use repeat airborne Light Detection and Ranging (LiDAR) surveys, with a variable temporal resolution (once every 5 months, on average) but targeted to capture the effects of major storm events, to quantify mass balance changes along the Hāpuku River in New Zealand (Fig. 1). The Hāpuku coseismic landslide was the dominant source of sediment to the valley in the wake of the Kaikōura earthquake (*36*), amounting to 6.6 million m^3^ delivered directly to the upper reaches of the river. A further 14.4 million m^3^ was deposited further upslope, making a total of 21 million m^3^ mobilized at this site immediately after the earthquake (*36*). The subsequent detailed capture of this sequence of impact and recovery provides a full longitudinal (downstream) picture of a mountain river’s dynamic reorganization in response to extreme overloading in the first 5 years after earthquake.

## RESULTS

**Fig. 2.**
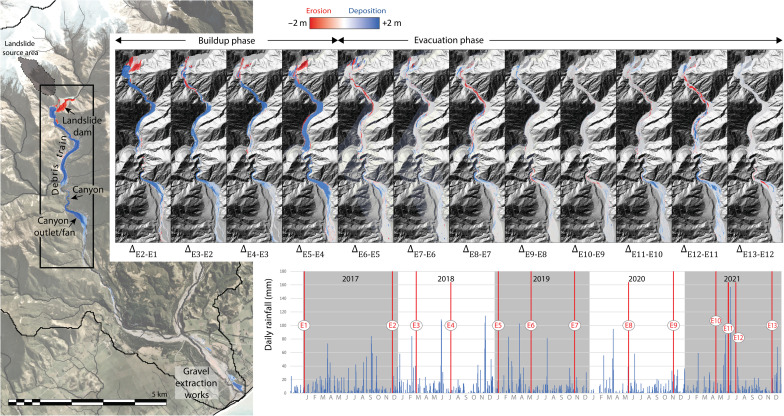
DEMs of difference. Difference models between survey digital elevation models (DEMs) highlight sites of erosion and deposition over 5 years since the Kaikōura earthquake. The first five surveys [four DEMs of difference (DoD)] show predominantly deposition downstream of the landslide deposit. In subsequent surveys, the river is shown to be cutting down through the valley train that built up in the upper valley. There is little morphological change evident in the lowermost reaches, other than industrial gravel extraction near the coast.

**Fig. 3.**
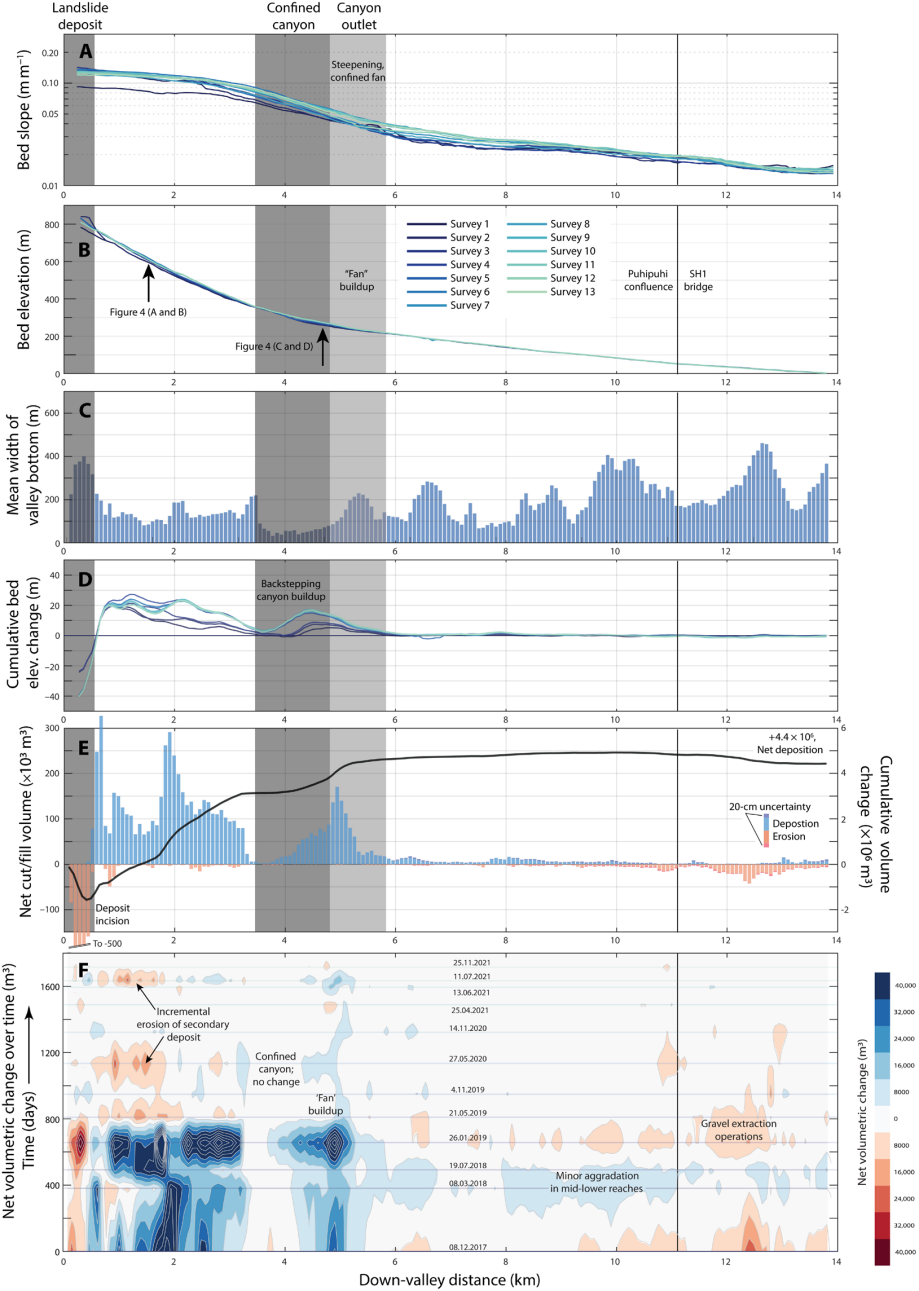
Longitudinal picture of change. Charts indicate (**A**) bed (thalweg) slope, traced using the D8 algorithm on each DEM (**B**) bed elevation, (**C**) average initial valley width, at 75 m intervals along the valley (**D**) cumulative change to bed elevation over time, (**E**) the net cut and fill volumes (first-to-final survey difference), downstream cumulative mass balance over all surveys, and (**F**) time-space diagram of net volumetric change (blue, accumulation; red, erosion). Values are interpolated from surveys (not from storm events), so the timing of changes is only indicative.

Figure 2 summarizes the changes to the river downstream from the landslide: Change models (Δ_E2-E1_ to Δ_E13-E12_) based on 13 airborne LiDAR surveys (epochs E1 to E13) reveal erosion (red) and deposition (blue) in the months and years following emplacement of the landslide deposit. The primary landslide dam deposit impounded a lake; this was initially eroded and redistributed through a combination of gravitational mass failure, debris flow, and some fluvial reworking over the course of several breach events (*36*). The April 2017 breach event lowered the lake level by at least 5 m. Erosion of this material, plus some additional material washed downstream from the landslide scar, fueled a rapid growth of the detrital train in the upper 3 km of the river. The upper channel was buried to a depth of 20 m by the time of E2, in December 2017, which includes a substantial dam breach event in September 2017. The wedge of aggradation extended roughly 5 km downstream (Fig. 3, E and F). The first four digital elevation models (DEMs) of difference (DoDs; between January 2017 and January 2019 (Δ_E2-E1_ to Δ_E5-E4_) show 1.61 million m^3^ of material eroding from the dam deposit, with two pulses delivering material downstream: the first episode ended some time before June 2018 (Δ_E4-E3_) (possibly during 3 to 14 April 2017 and/or 18 to 19 September 2017 storm events) and another following a 25 to 28 November 2018 storm event (Δ_E5-E4_). The total erosion of the dam deposit was 1.76 million m^3^.

Following survey E5 (~2 years after the earthquake), the river moved into a degradational phase. There was relatively little additional erosion of the landslide deposit, but erosion proceeded in the upper reaches of the valley. By November 2020 (E9), 1.2 million m^3^ of material had been removed from the upper debris train. Much of this was achieved in a March 2020 storm event (E8). A further 0.46 million m^3^ of material was mobilized from the upper reaches in flooding that occurred in June and July 2021. At the end of the surveys, a net total (Δ_E13-E1_) of 4.9 million m^3^ of weakly sorted debris flow and fluvial material had accumulated in the upper valley and mid-valley fan (Fig. 3E), amounting to roughly 23% of the assessed erosion from the primary Hāpuku landslide (including erosion of materials upslope). There were a number of other smaller landslides whose contributions to the upper valley fill have not been assessed here given their relatively minor volume. The volume of the in-valley landslide dam was reduced by 30%.

### Longitudinal trends

The change in thalweg elevation over the 5-year observation period after earthquake (Fig. 3D) shows that most of the buildup of sediment occurred in the upper reach between the site of the initial Hāpuku landslide deposit and roughly 2 to 3 km downstream (Fig. 3E). The slope of the upper channel after earthquake (initial 3.5 km from the landslide deposit) varies from 0.11 to 0.07 m m^−1^ (Fig. 3A).

Roughly 3.5 km downstream, the unnamed landslide-affected tributary joins the mainstem Hāpuku River and flows into a narrow bedrock canyon. The canyon is a relatively short component (just more than 1 km in length) of the longitudinal profile of the river. It has a distinctive narrow cross-sectional profile (30 to 40 m wide; Fig. 3C), contrasting markedly with the broader upstream and downstream segments. It is located at a hinge point within the longitudinal elevation gradient, where the valley slope transitions from >0.07 bed gradient in the upper section to the shallower (~0.03) lower valley reach. The mean gradient within the canyon is 0.06. There is no deposition in the upper canyon, and material is effectively funneled through to the lower valley, where it deposits into a confined fan. The deposit here builds up both as an advancing wedge moving downstream while also backstepping into the canyon upstream (Fig. 3, D and E).

### Hydraulic geometry and bed texture

**Fig. 4.**
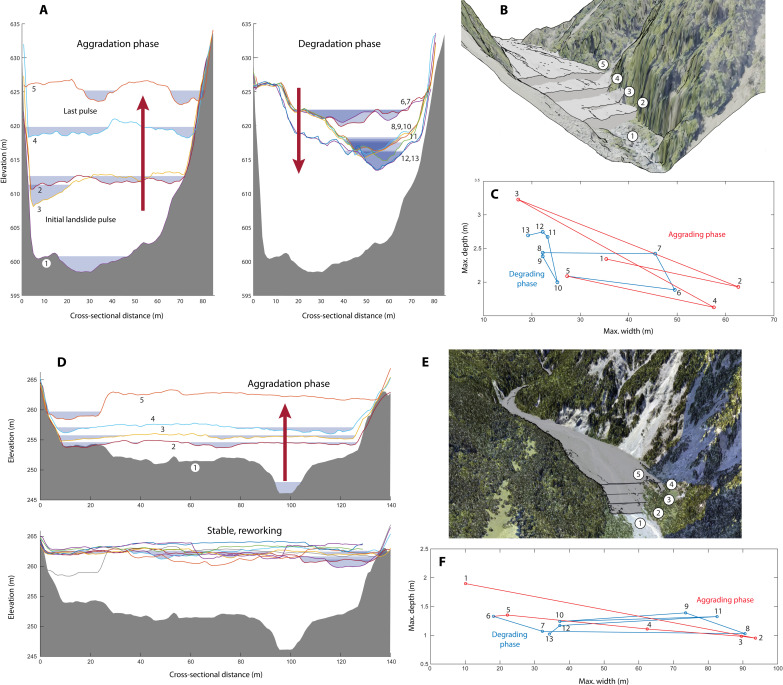
Changes to channel geometry and sediment storage. (**A**) Evolution of the channel cross section at 1.5 km from the landslide deposit. Bed elevation increases by 25 m following three pronounced aggradation episodes early in the event. The bed then drops by more than 10 m during subsequent degradation. (**B**) Morphology of the vertically building debris train in the first five surveys. (**C**) The width and depth of the active channel fluctuate considerably, but, generally, the channel widens during aggradation and narrows during degradation. (**D**) A confined fan near the downstream end of the canyon develops within the first available storage zone. (**E**) Morphology of the confined fan. (**F**) Channel morphology changes markedly, as wetted width expands from a 10-m channel to more than 90 m. This section does not see a sediment deficit and continues to avulse and aggrade. Sediment texture here is noticeably finer and better sorted than material in the valley upstream.

**Fig. 5.**
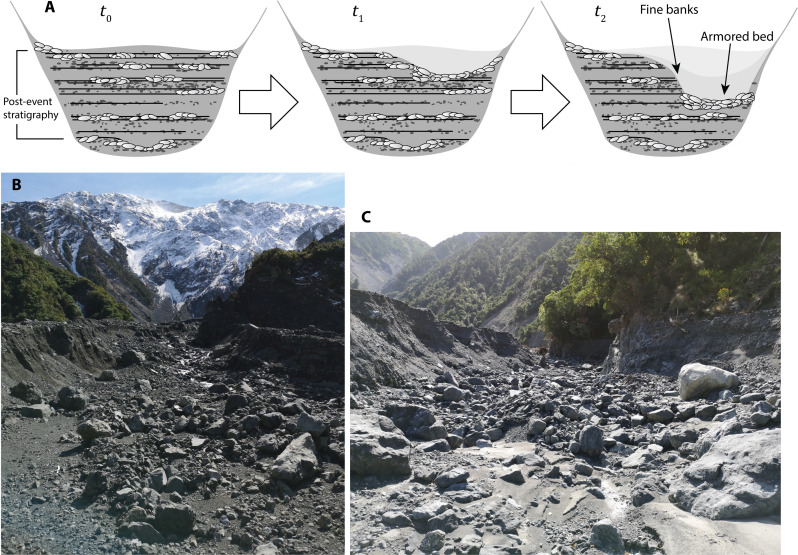
Channel condition during degradation. (**A**) Evolutionary sequence of armor development from channel incision into a poorly sorted valley fill. (**B**) Onset of degradation within the detrital train 1 km downstream from the landslide deposit. The upper banks of the incised channel span a distance of roughly 32 m. A very coarse lag deposit has evolved on the bed, while, by contrast, the bank consists mainly of loose, sandy material. The current channel is still some 15 m above the pre-disturbance elevation (see Fig. 4A). (**C**) At 2.5 km downstream, the channel has incised against the bedrock valley wall; it exhibits similar coarse-grained bed and fine-grained banks (channel width ≈ 27 m).

Figure 4A shows the sequence of buildup and downcutting for a cross section in the upper river. The pre-impact channel was a highly structured, step-pool channel whose bankfull aspect was relatively narrow and deep. The bed texture was characterized by boulders and cobbles, with a finer, mobile load transiting through. With the massive influx of poorly sorted sediment (boulder to sand fractions) following the earthquake, the river planform changed profoundly, becoming multi-threaded and highly unstable. With the valley deposit developing a slightly convex cross-sectional form (highest elevation at mid-valley), the principal thread of flow became attached to the valley wall in places, leading to some incision there during waning flows. Wetted channel cross sections (Fig. 4, A and C) show a pronounced trend of narrowing and deepening of the channel. With the diminution of readily mobilized material within the landslide deposit upstream, a phase of incisional downcutting began ~2 years after the earthquake, with the channel narrowing back to a wetted width that is narrower than the initial state. However, the phase of incisional narrowing only persisted for ~2.5 years before armoring of the bed by boulder size clasts (Fig. 5) sets in, and the channel began to widen.

Some 3.6 km downstream, below the canyon (Fig. 4D), a confined fan developed as a cobble-gravel mixture was deposited. As seen in Figs. 2 and 3F, the fan shows some signs of dynamic buildup in nearly every epoch regardless of whether erosion or deposition is predominant upstream. On the basis of observations from aerial photos and given consideration of the mass balance, materials may be sourced locally or from some distance above the canyon. Channel bankfull width more than doubles (Fig. 4, D and F), from 40 m to more than 80 m. Given the substantial remaining storage of sediment above the canyon (mid-valley), this lower reach has not yet “seen” the exhaustion of upstream sediment supply in the upper valley and continues to maintain a mostly aggradational regime.

## DISCUSSION

The aggradation of the Hāpuku River tributary under the influence of a major landslide delivery is a relatively extreme case of overloading a steepland river system. Repeat LiDAR and field surveys have revealed the post-earthquake cascade along the full length of the system, highlighting the importance of (i) feedbacks between sediment supply and evolving channel morphology, (ii) bed texture and armoring, and (iii) bedrock valley confinement, in mediating (in this case, delaying) the passage of the degradational wave of sediment evacuated from the landslide deposit.

The coevolution of alluvial channel geometry and the imposed sediment load emerge very clearly in the time series. The process of aggradation tends to promote greater storage in the reach, typically filling deeper channels and propagating channel widening. In turn, a wide, multichannel configuration involves shallower flow depth and relatively lower bed shear stress (*14*, *37*), leading to preferential onward transfer of finer fractions and deposition of distinctive sheet-flow bar and floodplain units. This process inevitably shapes the quantity and texture of the passing sediment load.

With reduced supply from upstream, the upper portion of the debris train undergoes some degree of erosional narrowing and deepening, recruiting local stores of material as it digs into the deposit (*38*, *39*). Two-dimensional numerical modeling has shown that channel narrowing can enhance transport capacity and allow rivers to evacuate sediment from landslides within a decade, but these models have limited representation of granular physics such as vertical segregation (*40*, *41*) and the incorporation of stratigraphy into the bed substrate (*11*, *42*). How this phenomenon translates to very coarse-grained, poorly sorted deposits in steep mountain valley settings has not been widely documented. The response in the Hāpuku River shows that channel armoring can become pronounced during this process (Fig. 5). Boulders selectively retained from the bounding heterogeneous sediments impart considerable structure and resilience to the degrading bed and act to diffuse erosive energy through hydraulic roughness. Second, the banks are typically sandy, non-cohesive, and unconsolidated (Fig. 5), so that it may be easier for the river to undermine them and widen the channel than it is to erode vertically into the bed.

The downstream pattern of valley width (Fig. 3C) has an evident impact on sediment storage and, ultimately, the response time of the system (Fig. 3E). If there is no accommodation space for broadening of the channel, then deep and erosive flows are maintained and material is flushed through. Conversely, with more room for floodplain storage and redistribution of sediment, the passage of an aggradational wave may be delayed. The mid-valley bedrock canyon provides no room for sediment storage, and thus the confined river rapidly conveys material to satisfy transport capacity. This leads to mainly “dispersive” movement of the landslide debris from the upper reaches (*43*–*45*), feeding the construction of the valley-confined fan downstream. Two substantial storm events in June and July 2021 have prompted erosion of the upstream deposit, but most of this mobilized material appears to have accumulated within this fan. Notably, there is only a relatively minor morphological imprint of the disturbance in the reaches downstream from this point, demonstrating the limited geomorphic impact of the landslide dam beyond about 6 km from the site of the primary landslide deposit, within the 5-year survey window. Gravel extraction was carried out in the lower, coastal reaches in anticipation of greatly heightened influx from upstream, but this has so far not materialized.

**Fig. 6.**
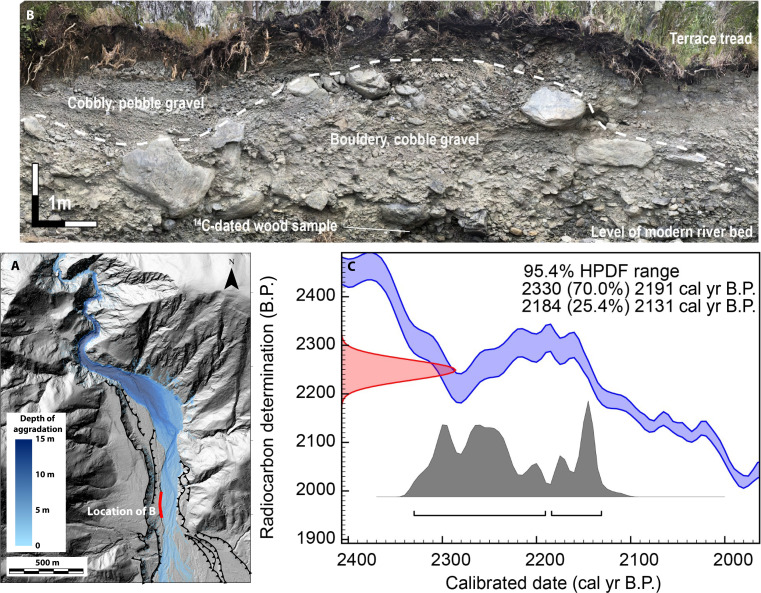
Sedimentary exposure of the current Hāpuku riverbank showing a long lived boulder gravel from a previous detrital train that is currently armoring the bed. (**A**) Location of the riverbank exposure at the toe of the confined fan that has accumulated after earthquake. (**B**) Structure form motion–derived orthomosaic of the riverbank sedimentary exposure showing a bouldery cobble gravel overlain by a pebble cobble gravel. (**C**) Calibrated radiocarbon age for a wood sample taken from the base of the exposure at the modern river level. cal yr B.P., calibrated years before the present; HPDF, highest probability density function.

The stratigraphy and chronology of alluvial terraces in the mountainous reaches of the Hāpuku River provide some insight into the longevity of landslide derived sediment trains. A stratigraphic exposure in the riverbank located at the toe of the confined fan shows that, at this location, the Hāpuku River was actively eroding a ~2200-year-old bouldery cobble gravel at the time of the earthquake (Fig. 6). The texture of this deposit is analogous to sediments now armoring the bed in the upper reach of the Kaikōura earthquake debris train, leading us to infer that the bouldery cobble gravel is a remnant from previous landslide-driven aggradation that has armored the riverbed for the last two millennia. The return time of similar episodes of earthquake-induced landsliding and sediment flux is likely to be substantially less because strong ground motions of PGA >0.5 g, equivalent to those caused by the Kaikōura earthquake in the Hāpuku River catchment, have a return time of just ~440 years based on the New Zealand National Seismic Hazard Model (*46*). Combined, these observations imply that remnants of past landslide-derived sediment trains may persist for time frames that exceed the inter-event time of earthquake-induced landsliding.

**Fig. 7.**
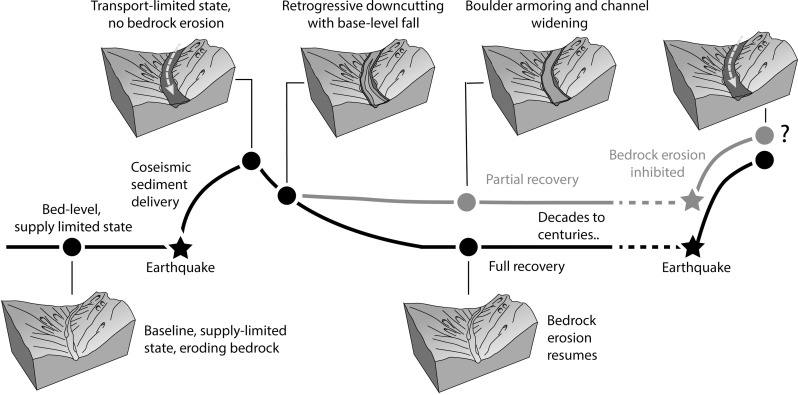
Timescale of evacuation and recovery, relative to the onset of future seismic events. The recurrence interval of earthquakes in the study area may be shorter than the timescale of evacuation of coarse-grained deposits from a given seismic event creating the potential for long-lived hiatuses in bedrock incision.

The potential for earthquake-driven sediment trains to armor the bed for time frames longer than the return time of their trigger mechanism provides a mechanism by which long-term hiatuses in bedrock incision can occur in active mountain belts. Such long-term hiatuses are required to explain observations of very high bedrock incision rates observed over short (*47*) or even event (*48*) timescales that are often too high to be sustained over geological timescales (Fig. 7) (*18*). They also provide an explanation for why rates of river incision are dependent on the measurement interval, complicating efforts to infer tectonic or climatic forcing from changes in river incision rate through time (*17*, *18*). Observations that bedrock channels in active mountain belts are often covered in sediment (*35*, *49*, *50*) also imply that long-term hiatuses in bedrock incision may be relatively commonplace.

Given the purview of traditionally resourced science programs is a few years at most, determining how mountain rivers evacuate earthquake-induced landslide sediment to maintain rates of bedrock incision will require numerical modeling. However, the complex patterns exhibited here point to improvements needed in model representations of mass flow processes in steep valleys, bank erosion dynamics, grain imbrication and armoring processes, differential transport formulations, and changing hydraulic geometry. The case of the Hāpuku is, of course, one scenario among a broad spectrum of possible topographic and sedimentary conditions; more sophisticated models will help to explore these. When these factors are incorporated into improved river morphodynamic models, it will become possible to improve estimates of evacuation times of coseismic landslide sediment from mountain catchments.

The time frame over which rivers work to evacuate sediment produced by earthquake-induced landslides controls the probability of aggradation and flood hazard to communities and infrastructure on range-front fans and floodplains (*19*, *51*). The same time frame is critical for understanding the spatiotemporal distribution of river incision into bedrock, for closing the mass balance of earthquakes and resolving their role in the long-term development of mountain belts (*10*, *16*, *46*, *52*). However, the results from this work emphasize that there are many important boundary conditions and contingencies that will lead to a wide distribution in possible evacuation times. For example, the feedback between sediment train incision, channel morphology, and armoring of the bed observed in the Hāpuku River suggests that enhanced sediment evacuation from channel narrowing may be relatively short-lived because armoring of the bed by boulders can promote increased channel roughness and enhanced likelihood of erosion of finer-grained banks, reducing stream power and slowing vertical incision into the deposit (Fig. 7). Hence, it may take decades or even centuries to fully evacuate sediment from landsliding triggered by the Kaikōura earthquake.

## MATERIALS AND METHODS

### Airborne LiDAR surveys

Differencing of high-resolution airborne LiDAR surveys was used to assess changes in sediment storage along the length of the Hāpuku River, in the 5 years since the earthquake. Following the landslide event, a regional LiDAR survey was flown over rivers and hillslopes in the zone of immediate shaking and impact. Twelve surveys were flown in subsequent months; the timing and details are outlined in Table 1. This database of topographic change provides important insights into the role of valley confinement, linkages with lateral sediment sources, sediment texture, and the residence time of the surplus landslide-derived sediment.

**Table 1.** Airborne LiDAR acquisition over the study area. ppm^2^, points per square meter.

Epoch	Date	Instrument	Point density	Notes
1	3 December 2016 to 6 January 2017	Riegl Q1560	2 to 5 ppm^2^	First regional survey following Kaikōura earthquake
2	1 to 8 December 2017	Riegl Q1560	2 to 5 ppm^2^	
3	8 March 2018	Leica ALS60	2 to 5 ppm^2^	
4	19 July 2018	Leica ALS60	2 to 5 ppm^2^	
5	26 to 31 January 2019	Optech Orion H300	4 ppm^2^	Second regional (catchment) survey
6	21 May 2019	Leica ALS60	4 ppm^2^	
7	3 to 4 November 2019	Leica ALS60	4 ppm^2^	
8	27 May 2020	Leica ALS60	4 ppm^2^	
9	14 November 2020	Leica ALS60	4 ppm^2^	
10	24 to 25 April 2021	Leica ALS60	4 ppm^2^	
11	10 to 13 June 2021	Leica ALS60	4 ppm^2^	After Canterbury floods
12	10 to 11 July 2021	Leica ALS60	4 ppm^2^	After local floods
13	25 November 2021	Leica ALS60	4 ppm^2^	

Surveys were carried out following roughly similar protocols. The precision of the survey points was estimated to be ±0.1 m (95% confidence interval). Outliers, vegetation, noise from water strikes, and other sources were filtered to generate a bare-earth point cloud that was then gridded to 1-m resolution rasters. A common grid extent and resolution was maintained for all surveys. Red Green Blue (RGB) photos with 0.1-m approximate ground sampling distance or better accompanied all surveys, such that changes observed in the elevation dataset could be confirmed with the imagery.

Difference maps were obtained by subtracting the bare-earth LiDAR rasters in a MATLAB environment. A uniform noise threshold of 20 cm was used as the typical two-sigma range for random errors for all change maps to reduce bias arising from noise within the datasets. This estimate represents the potential mean error over long reaches and is not an indication of point-level precision.

Longitudinal patterns of gross erosion, deposition, and net change were quantified using regular valley-spanning polygons of variable width but sectioned at 100-m intervals along the valley centerline. To reduce the possible influence of any uncorrected systematic errors, the area of analysis was truncated to exclude areas of the valley floor where both measured change and geomorphic position suggest no fluvial geomorphic change occurred.

Spatial patterns in sediment storage were visualized as the cumulative sum of net change, starting at the upstream edge of the DoD extent and moving downstream (“downstream cumulative net change”; Fig. 3D). Reaches of net erosion appear as a downward trending line, while net depositional reaches appear as upward trending lines.

Channel slope was measured by tracing the channel using a D8 flow-routing algorithm. The relatively high variation in slope values exhibited in lower reaches in Fig. 3A is likely due to the variable sinuosity of the channel within each slope measurement.

### Hydraulic geometry

To assess changing bankfull geometry in Fig. 4, cross-sectional flow estimates were generated using Manning’s equation, with channel roughness values computed according to assessment of local conditions: 0.040 in the very steep and coarse-grained section [0.08 gradient, Dsurf50 (median grain size of the surface gravel material) ≈ 64 mm] and 0.035 in the somewhat shallower slope section below the canyon (0.08 gradient, Dsurf50 ≈ 45 mm). Water surface level was changed iteratively until the equation was satisfied for the imposed discharge. The discharge imposed was based on an approximate estimate of high competent flood discharge in each reach (350 m^3^/s at the coast). The resulting width and depth values for each cross section are thus generated from the same governing conditions and are meant to be illustrative of the changing channel aspect (width/depth ratio) with changing sediment balance conditions in each reach.

### Radiocarbon dating

A fragment of a small tree trunk with a 4-cm-diameter branch attachment was located in situ at the base of a sedimentary exposure in the current Hāpuku riverbank (Fig. 7). A subsample of wood from the outermost rings of the branch attachment was taken for radiocarbon dating to avoid the potential for inbuilt age associated with the tree’s trunk. The wood sample was cleaned of residual sediment and pretreated using a cellulose extraction. The pretreated cellulose was converted to CO_2_ by combustion, graphitized, and measured by accelerator mass spectrometry at the Rafter Radiocarbon Laboratory (*53*). The conventional radiocarbon age was converted to calendar years using the ShCaL20 calibration curve (*54*) in the OxCal4.4 software (Table 2) (*55*).

**Table 2.** Conventional radiocarbon age (CRA) for the wood sample from the Hāpuku riverbank exposure. F(Mod), fraction modern.

Sample ID	Label code	F(Mod)	F(Mod) σ	CRA	CRA σ	δ^13^C	δ^13^C α
Hāpuku Terrace 1	NZA73885	0.75579	0.002038	2249	21	−24.46	0.2
